# Effects of height-induced postural threat on static and dynamic balance performance in healthy individuals: a systematic review with meta-analysis

**DOI:** 10.3389/fnhum.2025.1705947

**Published:** 2025-12-12

**Authors:** Anna Maria Wissmann, Thomas Muehlbauer, Mathew W. Hill

**Affiliations:** 1 Division of Movement and Training Sciences/Biomechanics of Sport, University of Duisburg-Essen, Essen, Germany; 2 School of Psychology and Vision Sciences, University of Leicester, Leicester, United Kingdom

**Keywords:** “stiffening” response, age, height-induced threat, postural control, standing, task difficulty, walking

## Abstract

**Background:**

Height-induced postural threat, such as standing or walking at elevation, elicits fear-related adaptations in balance control. Understanding these adaptations is crucial for interpreting motor behavior under anxiety and for informing rehabilitation and fall-prevention interventions. However, no previous systematic review with meta-analysis has quantified how height exposure influences balance performance.

**Objective:**

The objective was to aggregate, characterize, and quantify the effects of height-related postural threat on static and dynamic balance performance.

**Methods:**

A systematic literature search in the electronic databases PubMed, Web of Science, and SPORTDiscus was conducted from their inception date until 15 September 2025. Eligible cross-sectional studies compared ground-level (*no threat*) versus elevated (*threat*) conditions in healthy participants. Static balance outcomes during upright stance included sway amplitude and frequency; dynamic balance outcomes while walking included gait velocity. Included studies were coded for gender, age, postural threat conditions, balance assessment/outcome, and test modality. Methodological study quality/design and risk of bias was assessed using the Appraisal tool for Cross-Sectional Studies. Heterogeneity was quantified using *I^2^* statistics, and sensitivity was evaluated via Leave-One-Out method. Standardized mean differences (*SMD*) were calculated and analyses were stratified by age group (i.e., children, young adults, older adults).

**Results:**

The search identified a total of *N* = 438 records, and 25 of them (involving a total of 877 participants) met the inclusion criteria. Concerning static balance (18 studies, 44 comparisons), postural threat resulted in small-sized (*SMD* = 0.20) decreases in sway amplitude measures and in large-sized (*SMD* = 1.06) increases in sway frequency measures, indicating a potentially protective “stiffening” response. However, children did not use the “stiffening” response when standing at height (sway amplitude: *SMD* = −0.41; sway frequency: *SMD* = −0.04). Regarding dynamic balance (7 studies, 16 comparisons), postural threat led to large-sized (*SMD* = 1.33) declines in gait velocity, and this was more pronounced for conditions with a high (*SMD* = 1.78) than a low (*SMD* = 1.05) difficulty level.

**Conclusion:**

Height-induced postural threat evoked functional changes in static (i.e., decrease/increase in sway amplitude/frequency measures) and dynamic (i.e., decrease in walking speed measures) postural control. For static balance this is indicative of an effective “stiffening” response which is apparently not yet developed in children. For the dynamic balance, the further decrease in gait velocity during difficult walking conditions at height implies a compensatory mechanism to increase stability. Despite consistent direction and magnitude of effects, substantial between-study heterogeneity limits the generalizability of these findings, and results should therefore be interpreted with caution.

## 1 Introduction

It is firmly established that negative emotional states, such as anxiety (i.e., anticipation of future uncertainty; Harris et al., 2023) and fear (i.e., immediate response to an imminent or present threat; Ellmers et al., 2020b), can disrupt balance behavior (Adkin and Carpenter, 2018). Empirical support for this notion is drawn largely from studies experimentally altering emotional states (i.e., fear) within controlled laboratory environments (see Adkin and Carpenter, 2018, for review). This is most frequently achieved by raising the height of the surface on which individuals stand/walk, which serves to threaten balance safety by introducing the potential for injurious consequences should a loss of balance occur (Sturnieks et al., 2016; Hill et al., 2024a). The imposed threat to postural control associated with the manipulation of environmental context has been shown to evoke elevated fear/anxiety and lower confidence in balance, leading to behavioral adaptations which may, paradoxically, increase the risk of falling (Hill et al., 2023; Hill et al., 2024a; Lambrich et al., 2025).

A number of studies (Carpenter et al., 1999, 2006; Brown et al., 2002a) have shown that the central nervous system (CNS) imposes modifications to balance behavior during height-induced postural threat conditions. For example, when standing quietly at height, fearful individuals exhibited higher-frequency center of pressure (CoP) adjustments, often accompanied by reductions in CoP displacement amplitude (Carpenter et al., 1999, 2006). These changes have been interpreted as part of a postural “stiffening” response (Carpenter et al., 2001; Brown et al., 2006), where increased stiffness of the ankle joint—mediated by greater co-contraction of lower leg muscles—serves to tighten control of the center of mass (CoM) within the limits of the base-of-support (BoS) (Young and Mark Williams, 2015). Under dynamic conditions, fearful individuals tend to adopt a more cautious gait pattern, characterized by reduced walking velocity, shorter step length, a widened BoS, and increased duration of double-limb support during gait at height (Brown et al., 2002a; McKenzie and Brown, 2004; Delbaere et al., 2009; Ellmers et al., 2020a; Lambrich et al., 2025). Although a narrative review of height-induced postural threats has been previously published (Adkin and Carpenter, 2018), a systematic review and meta-analysis is warranted to address between-study variability. Such an approach would allow for a more rigorous and comprehensive synthesis of the evidence regarding the impact of height-induced threats on balance behavior.

More precisely, our current understanding of modifications in balance behavior due to height-induced postural threat is primarily derived from studies involving young adults (Brown and Frank, 1997; Carpenter et al., 2001; Lelard et al., 2014; Ellmers and Young, 2018; Ellmers et al., 2020c; Guo et al., 2023; Hill et al., 2023; Hill et al., 2024b), with relatively few investigations examining whether postural responses to height-induced threat differ by age (Brown et al., 2002b; McKenzie and Brown, 2004; Laufer et al., 2006; Sturnieks et al., 2016; Hill et al., 2024a). Nevertheless, broader research examining age differences in postural responses to height-induced threat indicates that older adults typically demonstrate heightened emotional responses and altered balance control relative to young adults (Brown et al., 2006; Carpenter et al., 2006; Johnson et al., 2019a; Johnson et al., 2019b; Hill et al., 2024a). In addition, children compared to adults exhibit less mature postural control mechanisms and heightened emotional responses to environmental challenges. Specifically, studies have shown that height-induced postural threat elicit greater sway variability and stronger physiological arousal in children than in adults (e.g., Omorczyk et al., 2021; Wissmann et al., 2025a). Such developmental differences justify the inclusion of children in this analysis to better understand age-specific adaptations to height-induced postural threat. Collectively, despite the fact that there is substantial evidence regarding age differences in postural responses to height-induced threat, a comprehensive synthesis remains necessary to elucidate the magnitude and nature of these age-related differences in postural control and physiological reactivity.

Moreover, the difficulty of the postural task itself may also modulate behavioral and emotional responses to height-induced postural threat (Tokuno et al., 2018). More demanding balance tasks—such as altering the support surface (i.e., foam or reducing the size of the BOS) or removing sensory information (e.g., standing with eyes closed)—may amplify the effects of height-induced postural threat by increasing attentional demands and challenging the capacity to maintain stability. These increased demands may further heighten fear and anxiety, leading to exaggerated postural stiffening or insufficiently adjusted gait modifications that could elevate fall risk. Conversely, task difficulty may interact with individual differences in balance confidence, such that individuals with greater balance capacity can better mitigate threat-induced changes in behavior (Adkin and Carpenter, 2018; Carpenter et al., 2001; Hill et al., 2024a). Systematically evaluating how task demands influence postural threat responses across different populations is critical for refining our understanding of balance adaptations and informing targeted interventions for individuals at greater risk of falls (Davis et al., 2009; Stins et al., 2009).

While individual studies have demonstrated that height exposure alters balance control, no meta-analysis has systematically quantified these effects across age groups and task conditions. The present study therefore provides a quantitative synthesis to clarify the magnitude and direction of height-induced adaptations in both static and dynamic balance. Exposure to height during standing or walking triggers fear-related adjustments in postural control. Gaining insight into these responses is crucial to understand motor behavior under conditions of anxiety and to inform rehabilitation strategies and fall-prevention programs. Thus, the present study was aimed to compliment the previous narrative review by Adkin and Carpenter (2018), characterizing, summarizing, and quantifying behavioral differences in measures of static and dynamic balance between different height-related threat conditions (i.e., *no threat* = ground-level *vs. threat* = above ground-level) with varying levels of task difficulty in healthy individuals of different ages. Based on the above-mentioned findings we hypothesized that standing at height would lead to a postural “stiffening” response, i.e., a decrease in sway amplitude and an increase in sway frequency measures, while walking at height would result in a decrease in walking speed measures. Furthermore, the aforementioned changes would be more pronounced for tasks with a high (e.g., standing with eyes closed or dual-task walking) compared to a low (e.g., standing with eyes open or single-task walking) difficulty level. Contrary to the expected “stiffening” response in adults, we expected different adaptions to height-induced postural threat in children. Specifically, due to immaturities in postural control mechanisms (Riach and Hayes, 1987; Steindl et al., 2006), children should exhibit an ineffective postural adaptation strategy when standing (i.e., increase/decrease in sway amplitude/frequency) and walking (e.g., no reduction in gait velocity) above versus on ground-level (Hill et al., 2024a; Wissmann et al., 2025b).

## 2 Methods

### 2.1 Literature search

**Figure 1:**
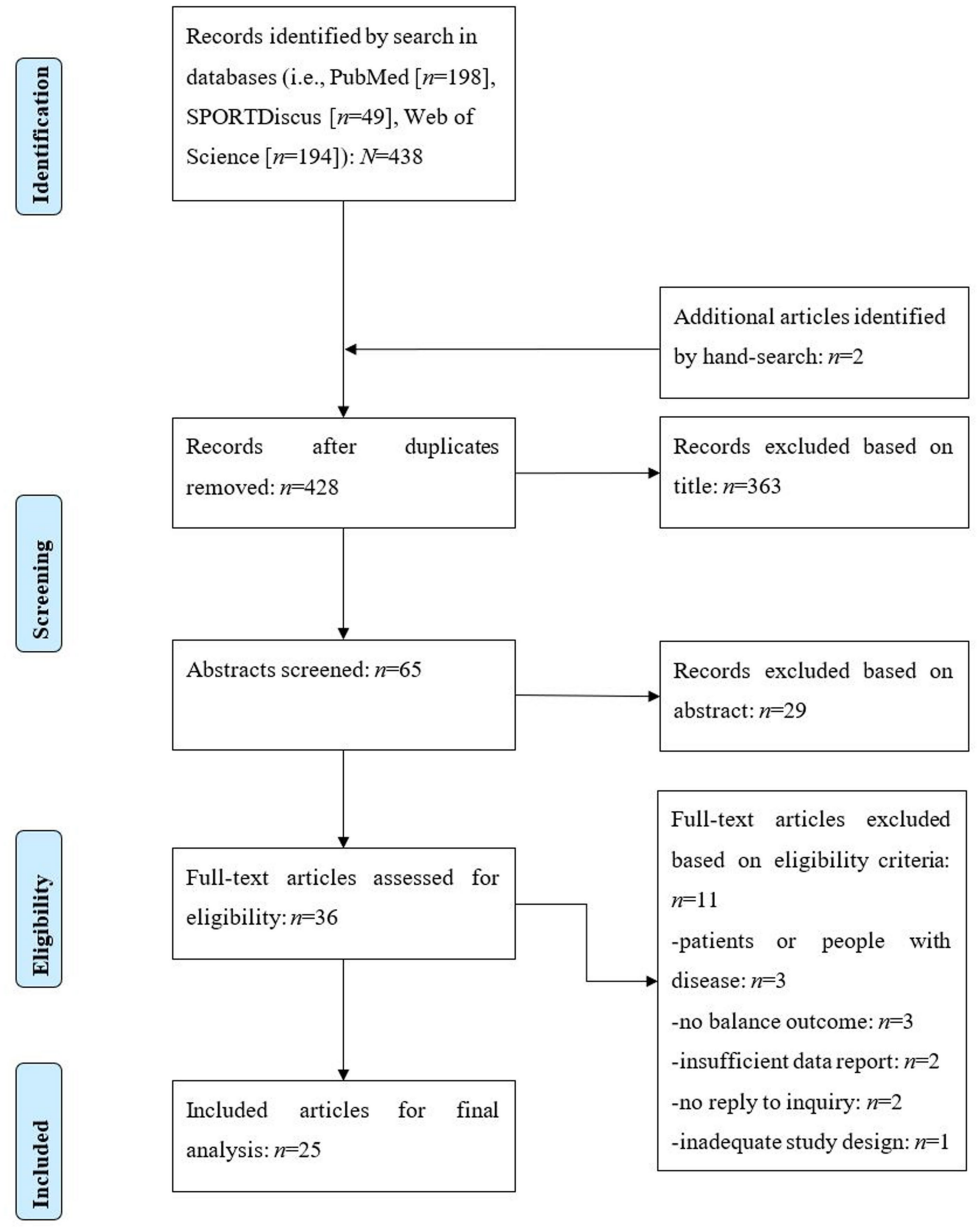
PRISMA flow chart illustrating the different phases of literature search.

The systematic literature search was performed in the electronic databases PubMed, Web of Science, and SPORTDiscus using the following Boolean search strategy: (((“balance performance” OR “postural control” OR “postural balance” OR “postural stability” OR “balance test” OR “balance function” OR “balance ability”) AND (“threat” OR “surface height” OR “raised surface”) NOT (patient OR disease OR impairment OR dysfunction OR disability OR pathology))). For all databases, the article search was conducted from their inception date until 15 September 2025. We included only articles that were written in English language with full-text access and investigated humans. In addition, the reference lists of the included articles were screened for other studies potentially suitable for inclusion. After removing all duplicates, the title and abstract of all articles were checked for eligibility according to the inclusion and exclusion criteria. The full texts of all potentially eligible records were independently assessed by two authors (AMW and TM) and disagreement was resolved through discussion and consent. The different phases of literature search consisting of identification, screening, eligibility, inclusion and exclusion of records are presented in Figure 1 by using the PRISMA flow chart (Moher et al., 2009). Furthermore, the PRISMA checklist (Moher et al., 2009) was used for transparent reporting of systematic reviews and meta-analyses (Supplementary Table S1). The protocol was not prospectively registered in PROSPERO or a similar database prior to conducting the systematic review because it was a research-based learning project with a very short duration that required a rapid evidence assessment. To promote reproducibility, the data extraction spreadsheet is provided in the Supplementary Table S2.

### 2.2 Criteria for study selection

To be eligible for inclusion, studies had to meet the following criteria: (a) subjects were healthy; (b) studies reported a “threat” (i.e., above ground-level) and a “no threat” condition (i.e., ground-level); (c) at least one balance category (static or dynamic) was assessed; (d) descriptive statistics (e.g., mean value and standard deviation) were reported; (e) a cross-sectional study design was used. Studies were excluded if: (a) patients or people with disease were studied; (b) studies reported different “threat” (e.g., low *vs.* medium *vs.* high) but not a “no threat” condition (i.e., ground-level); (c) the reported outcomes did not include a measure of balance performance; (d) the described data did not allow the calculation of effect size; (e) study authors did not reply to our inquiry to send original data by email; (f) an intervention study was conducted but no baseline data were reported; (g) virtual-reality height exposures were used.

### 2.3 Classification of studies

**Figure 2:**
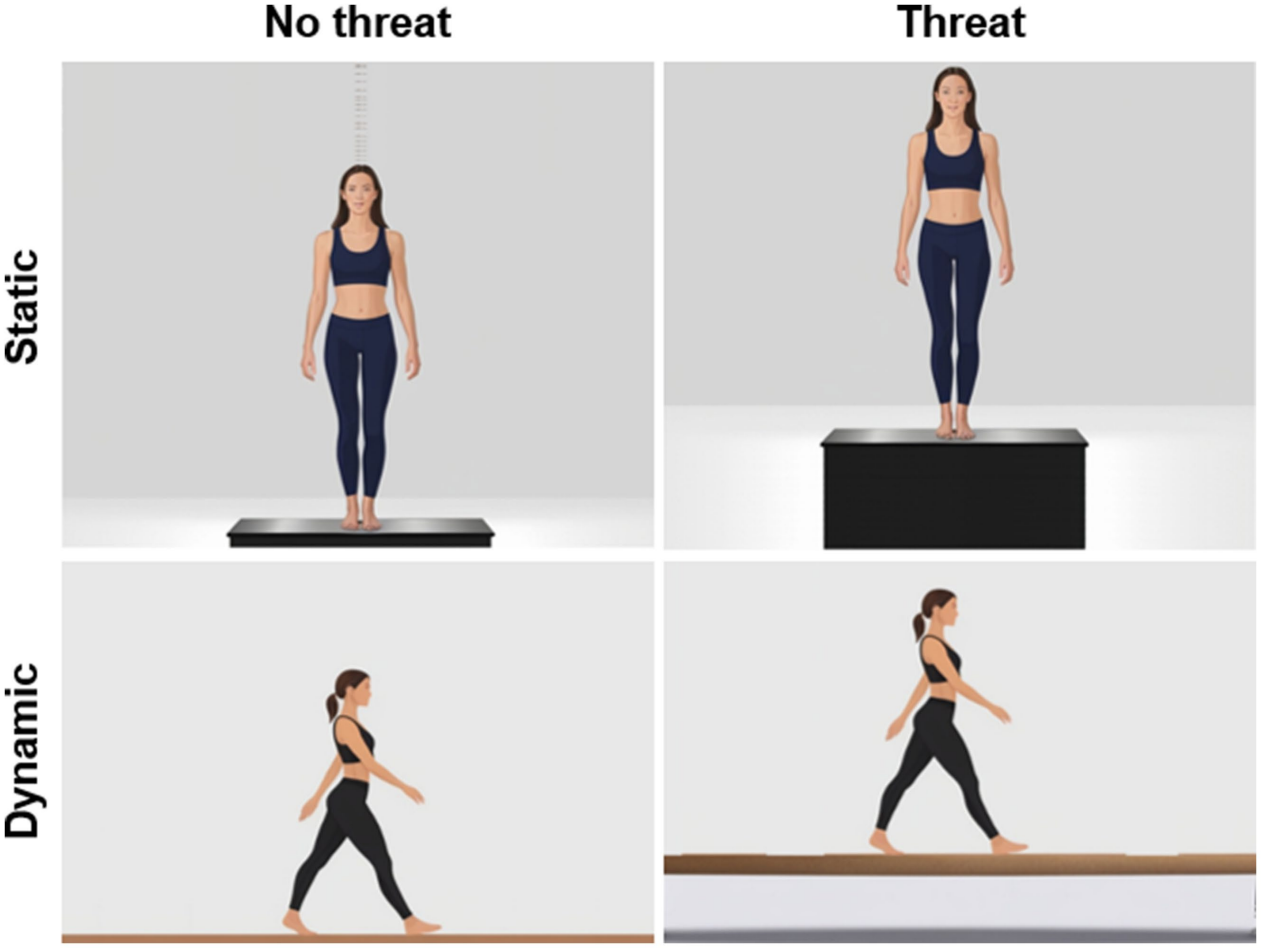
Schematic diagram of the different postural threat conditions (no threat = ground-level vs. threat = above ground-level) used during the assessment of static (top) and dynamic (bottom) balance performance.

We classified the included studies using the following criteria: authors, year of publication, number of subjects, gender (i.e., female, male), age (i.e., children: 6–12 years, young adults: 18–64 years, older adults: ≥65 years), height-induced postural threat conditions (i.e., ground-level, above ground-level, Figure 2), balance assessment and outcome by category (static or dynamic), test modality (i.e., task difficulty). In accordance with Shumway-Cook and Woollacott (Shumway-Cook and Woollacott, 2016), balance performance was classified into *static* (i.e., maintenance of a steady position while standing) and *dynamic* (i.e., maintenance of a steady position while ambulation) postural control. In terms of static balance, residual CoP amplitude (mm or cm or m) and frequency (Hz) measures were used for further analyses. A reduction in CoP amplitude combined with an increase in CoP frequency was considered to be an effective “stiffening” response. Regarding dynamic balance, gait velocity (m/s) was used for further analyses.

### 2.4 Appraisal of methodological study quality

Methodological study quality was rated by using the Appraisal tool for Cross-Sectional Studies (Downes et al., 2016). The tool includes of 20 questions addressing study quality, study design, and risk of bias, which have to be answered with “yes,” “no,” or “do not know” (Supplementary Table S3). Seven questions (1, 4, 10, 11, 12, 16, and 18) refer to the quality of reporting and another seven questions (2, 3, 5, 8, 17, 19, and 20) to the quality of the study design. Additional six questions (6, 7, 9, 13, 14, and 15) relate to a possible risk of bias. Further three questions (7, 13, and 14) were excluded from the analysis because they address aspects (e.g., potential non-responders) that were not applicable for the included studies. The assessment of methodological study quality was independently performed by two authors (AMW and TM) and disagreement was resolved through discussion and consent.

### 2.5 Statistical analyses

To quantify changes in static and dynamic balance performance between ground level (no threat) and height level (threat) conditions, the within-subject weighted standardized mean difference (*SMD*) – a measure of effect size – was calculated using the following formula:

$$\begin{array}{l} \mathrm{SMD}=(-1)-(\text{threat mean value}–\mathrm{no}\;\text{threat mean value})\\ /\text{pooled standard deviation} \end{array}$$

Using JASP version 0.19.2. Depending on the outcome measure, *SMD* values can be negative or positive. Therefore, balance outcomes that indicate a postural “stiffening” response (i.e., decrease in sway amplitude measures/increase in frequency measures) or a decrease in gait velocity were given a positive sign. Conversely, a negative *SMD* value means an insufficiently adapted and ineffective postural adaptation strategy. As suggested by Cohen (Cohen, 1988), *SMD* values were classified as small (0 ≤ 0.49), moderate (0.50 ≤ 0.79), or large (≥ 0.80). Furthermore, heterogeneity (*I*^2^) between studies was computed by using the following formula (Deeks et al., 2008):

$$I^{2}=(Q–\mathrm{df}/Q)\;100\%$$

Where *Q* is the *Chi^2^* statistics and *df* represents the degrees of freedom (Higgins and Thompson, 2002). In accordance with Deeks and colleagues (Deeks et al., 2008), *I*^2^ value were classified as trivial (0 ≤ 40%), moderate (30 ≤ 60%), substantial (50 ≤ 90%), or considerable (75 ≤ 100%) heterogeneity. If a considerable *I^2^* value was reached, age-specific subgroup analyses were performed. In addition, the higher classification level was determined and, in cases of considerable or significant heterogeneity, the leave-one-out (LOO) method (Harrer et al., 2021) was applied to check whether the *I^2^* value could be reduced.

## 3 Results

Figure 1 shows the different phases of the systematic electronic literature search, which resulted in a total of 438 records. After removing duplicates, screening titles, reading abstracts, and excluding ineligible articles, 25 studies were included in the present meta-analysis.

### 3.1 Characteristics of the included studies

Table 1 shows the characteristics of the 25 included studies that investigated the effect of height-induced postural threat on static and dynamic balance performance in healthy individuals. Eighteen (Brown and Frank, 1997; Carpenter et al., 2001; Laufer et al., 2006; Huffman et al., 2009; Pasman et al., 2011; Yiou et al., 2011; Lelard et al., 2014; Cleworth and Carpenter, 2016; Sturnieks et al., 2016; Ellmers et al., 2020c; Omorczyk et al., 2021; Ellmers et al., 2022; Guo et al., 2023; Hill et al., 2023; Hill et al., 2024a; Hill et al., 2024b; Tashiro et al., 2024; Wissmann et al., 2025a) and seven (Brown et al., 2002a; Gage et al., 2003; McKenzie and Brown, 2004; Ellmers and Young, 2018; Kluft et al., 2020; Lambrich et al., 2025; Wissmann et al., 2025b) studies reported variables of static and dynamic balance, respectively. In total, 877 subjects (*n* = 459 females) participated in the 25 studies, with four (Omorczyk et al., 2021; Hill et al., 2024a; Wissmann et al., 2025b) (a), twenty (Brown et al., 2002a; Brown and Frank, 1997; Carpenter et al., 2001; Gage et al., 2003; McKenzie and Brown, 2004; Laufer et al., 2006; Huffman et al., 2009; Yiou et al., 2011; Lelard et al., 2014; Cleworth and Carpenter, 2016; Sturnieks et al., 2016; Ellmers and Young, 2018; Ellmers et al., 2020c; Guo et al., 2023; Hill et al., 2023; Hill et al., 2024a; Hill et al., 2024b; Lambrich et al., 2025; Wissmann et al., 2025b) (a), and nine (Gage et al., 2003; McKenzie and Brown, 2004; Laufer et al., 2006; Pasman et al., 2011; Sturnieks et al., 2016; Kluft et al., 2020; Ellmers et al., 2022; Hill et al., 2024a; Tashiro et al., 2024) studies investigating children, young and older adults, respectively.

**Table 1:** Alphabetical overview of the included studies investigating the effect of height-induced postural threat on static and dynamic balance performance in healthy individuals.

References	No. of subjects; gender; age (mean ± SD or range)	Postural threat conditions	Assessment (outcome [unit]) by balance category	Test modality
Static balance				
Brown and Frank (1997)	8; F (0), M (8); 23.0 ± 5.7 years	No threat (ground-level) *vs.* threat (above ground-level: 76 cm)	*Static balance:* Bipedal stance without a safety harness on a force plate (**CoM displacement [m]**, velocity [m/s], position relative to ankle [m], latency to peak velocity [m/s], position [cm])	*N/A*
Carpenter et al. (2001)	8; F (4), M (4); 18–30 years	No threat (ground-level) *vs.* threat (above ground-level: 81 cm)	*Static balance:* Bipedal stance with a safety harness on a force plate (**CoP** and **CoM** **displacement [cm], frequency [Hz]**)	Standing at surface center and edge
Cleworth and Carpenter (2016)	20; F (*N/A*), M (*N/A*); 20.9 ± 2.1 years	No threat (ground-level) *vs.* threat (above ground-level: 320 cm)	*Static balance*: Bipedal stance with a safety harness on a force plate (**CoP displacement [cm], frequency [Hz]**)	
Ellmers et al. (2020c)	16; F (8), M (8); 25.9 ± 2.9 years	No threat (ground-level) *vs.* threat (above ground-level: 110 cm)	*Static balance:* Step task (gait initiation) without a safety harness on a force plate (**CoP displacement [cm]**; step length [cm]; APA latency (ms), APA efficiency [AU])	*N/A*
Ellmers et al. (2022)	44; F (31), M (13); 73.9 ± 7.0 years	No threat (ground-level) *vs.* threat (above ground-level: 60 cm)	*Static balance:* Bipedal narrow-stance without a safety harness on a force plate (**CoP displacement** [**mm], frequency [Hz]**)	*N/A*
Guo et al. (2023)	30; F (0), M (30); 23.9 ± 1.2 years	No threat (ground-level) *vs.* threat 1 (above ground-level: 5 cm) *vs.* threat 2 (above ground-level: 15 cm) v*s.* threat 3 (above ground-level: 25 cm)	*Static balance:* Bipedal and unipedal stance without a safety harness while wearing pressure-detecting insoles (**CoP displacement [mm]**, velocity [mm/s], time [s])	Bipedal and unipedal stance with eyes open and closed
Hill et al. (2023)	30; F (12), M (18); 22.0 ± 4.0 years	No threat (ground-level) *vs.* threat (above ground-level: 80 cm)	*Static balance:* Tandem stance without a safety harness on a force plate (**CoP amplitude [mm]**, **frequency [Hz]**)	Standing with arms free and restricted
Hill et al. (2024a)	38; F (17), M (21); 9.7 ± 0.8 years	No threat (ground-level) *vs.* threat (above ground-level: 80 cm)	*Static balance:* Bipedal stance without a safety harness on a force plate (**CoP amplitude [mm]**, frequency [Hz])	*N/A*
	45; F (19), M (26); 21.8 ± 4.0 years			
	15; F (7), M (8); 73.3 ± 5.0 years			
Hill et al. (2024b)	20; F (7); M (13); 21.3 ± 2.7 years	No threat (ground-level) *vs.* threat (above ground-level: 80 cm)	*Static balance:* Bipedal stance without a safety harness on a force plate (**CoP amplitude [mm]**, **frequency [Hz]**)	Standing with no or slow false or fast false HR feedback
	19; F (7); M (12); 21.5 ± 2.6 years			
	20; F (5), M (15); 25.9 ± 6.2 years			
Huffman et al. (2009)	48; F (24), M (24); 24.8 ± 3.9 years	No threat (ground-level) *vs.* threat (above ground-level: 320 cm)	*Static balance*: Bipedal stance with a safety harness on a force plate (**CoP displacement [cm], frequency [Hz]**)	*N/A*
Laufer et al. (2006)	20; F (11), M (9); 21.5 ± 3.7 years	No threat (ground-level) *vs.* threat (above ground-level: 85 cm)	*Static balance:* Bipedal stance without a safety harness on a force plate (**COP displacement [mm], frequency [Hz]**, velocity [cm/s])	Standing with eyes open and closed
	60; F (43), M (17); 77.5 ± 4.4 years			
Lelard et al. (2014)	32; F (17), M (15); 21.4 ± 2.3 years	No threat (ground-level) *vs.* threat (above ground-level: 80 cm)	*Static balance:* Bipedal stance without a safety harness on a force plate (**CoP displacement [mm]**, area [mm^2^])	*N/A*
Omorczyk et al. (2021)	34; F (34), M (0): 8–12 years	No threat (ground-level) *vs.* threat (above ground-level: 125 cm)	*Static balance:* Bipedal and tandem stance without a safety harness on a force plate (**COP displacement [mm], frequency [Hz]**, area [mm^2^])	Bipedal and tandem stance
Pasman et al. (2011)	16; F (7) M (9); 68.8 ± 5 years	No threat (ground-level) *vs.* threat 1 (above ground-level: 80 cm) *vs.* threat 2 (above ground-level: 160 cm)	*Static balance:* Bipedal stance with a safety harness on a force plate (**CoP displacement [mm], frequency [Hz]**)	*N/A*
Sturnieks et al. (2016)	9; F (4), M (5); 31 ± 5 years	No threat (ground-level) *vs.* threat (above ground-level: 65 cm)	*Static balance:* Bipedal stance without a safety harness on a force plate (**CoP displacement [mm], frequency [Hz]**)	*N/A*
	48; F (26), M (22); 76 ± 5 years			
Tashiro et al. (2024)	27; F (23), M (4); 77.9 ± 6.2 years	No threat (ground-level) *vs.* threat (above ground-level: 60 cm)	*Static balance:* Bipedal stance without a safety harness on a force plate (**CoP displacement [mm], velocity** [mm/s])	*N/A*
Wissmann et al. (2025a)	26; F (13), M (13); 9.8 ± 0.6 years	No threat (ground-level) *vs.* threat (above ground-level: 80 cm)	*Static balance:* Tandem stance without a safety harness on a force plate (**CoP amplitude [mm]**, **frequency [Hz]**, velocity [mm/s])	Standing with arms free and restricted
	23; F (11), M (12); 24.7 ± 4.0 years			
Yiou et al. (2011)	10; F (4), M (6); 29.7 ± 6.2 years	No threat (ground-level) *vs.* threat (above ground-level: 60 cm)	*Static balance:* Bipedal stance without a safety harness on a force plate (**CoG displacement [m]**, velocity [mm/s]; APA duration [s])	*N/A*
Dynamic balance				
Brown et al. (2002a)	16; F (8), M (8); 22.4 ± 2.3 years	No threat (ground-level) *vs.* threat (above ground-level: 60 cm)	*Dynamic balance:* Walking with a safety harness along a 7.2-m path (**gait velocity [m/s]**; stride length [cm]; angular displacement [°], velocity [°/s])	Walking over different beam widths (i.e., 15 cm [constrained] and 60 cm [unconstrained])
	15; F (12), M (3); 67.5 ± 3.9 years			
Ellmers and Young (2018)	15; F (7), M (8); 25.5 ± 2.5 years	No threat (ground-level) *vs.* threat (above ground-level: 110 cm)	*Dynamic balance:* Walking without a safety harness along a 3.3-m path and performing a precision stepping task (**gait velocity [m/s]**; stepping accuracy [mm])	Walking without and with a concurrent cognitive task (i.e., arithmetic calculation)
Gage et al. (2003)	16; F (8), M (8); 22.4 ± 2.3 years	No threat (ground-level) *vs.* threat (above ground-level: 60 cm)	*Dynamic balance:* Walking with a safety harness along a 7.2-m path (**CoM velocity [m/s]**; stride length [cm]; single−/double-limb stance duration [%])	Walking over different beam widths (i.e., 15 cm [constrained] and 60 cm [unconstrained])
	15; F (12), M (3): 67.5 ± 3.9 years			
Kluft et al. (2020)	24; F (14), M (10); 73.8 ± 4.9 years	No threat (ground-level) *vs.* threat (above ground-level: 78 cm)	*Dynamic balance:* Walking with a safety harness along a 4.0-m path and performing a decent stepping task (**gait velocity [m/s]**; step length [m])	*N/A*
Lambrich et al. (2025)	22; F (11), M (11); 24.4 ± 4.9 years	No threat (ground-level) *vs.* threat (above ground-level: 80 cm)	*Dynamic balance:* Walking without a safety harness along a 5.0-m balance beam (**gait velocity [m/s]**; cadence [n/s]; step time [s]; impulse [Ns])	Walking with arms free and restricted
McKenzie and Brown (2004)	15; F (10), M (5); 22.5 ± 2.8 years	No threat (ground-level) *vs.* threat (above ground-level: 60 cm)	*Dynamic balance:* Walking with a safety harness along a 7.2-m path (**lead limb velocity [m/s]**; trail crossing velocity [m/s]; whole-body CoM velocity[m/s])	Walking over different beam widths (i.e., 15 cm [constrained] and 60 cm [unconstrained])
	17; F (10), M (7): 68.9 ± 4.9 years			
Wissmann et al. (2025b)	29; F (18), M (11); 11.1 ± 0.3 years	No threat (ground-level) *vs.* threat (above ground-level: 80 cm)	*Dynamic balance:* Walking without a safety harness along a 5.0-m balance beam (**gait velocity [m/s]**; cadence [n/s])	Walking with arms free and restricted
	26; F (15), M (11); 24.0 ± 4.7 years			

Bold outcomes were included in the quantitative meta-analysis. APA anticipatory postural adjustment, AU arbitrary unit, CoG center of gravity, CoM center of mass, CoP center of pressure, F female, HR heart rate, M male, N/A not applicable.

### 3.2 Methodological quality of the included studies

The assessment of methodological study quality according to the Appraisal tool for Cross-Sectional Studies revealed that most included studies met the criteria for study quality, study design, and risk of bias (Supplementary Table S3). Precisely, all included studies fulfilled ≥ 4 out of 7 criteria regarding quality of reporting and study design. Furthermore, all included studies fulfilled ≥2 out of 3 criteria with respect to risk of bias.

### 3.3 Effect of height-induced postural threat on static balance performance

**Figure 3:**
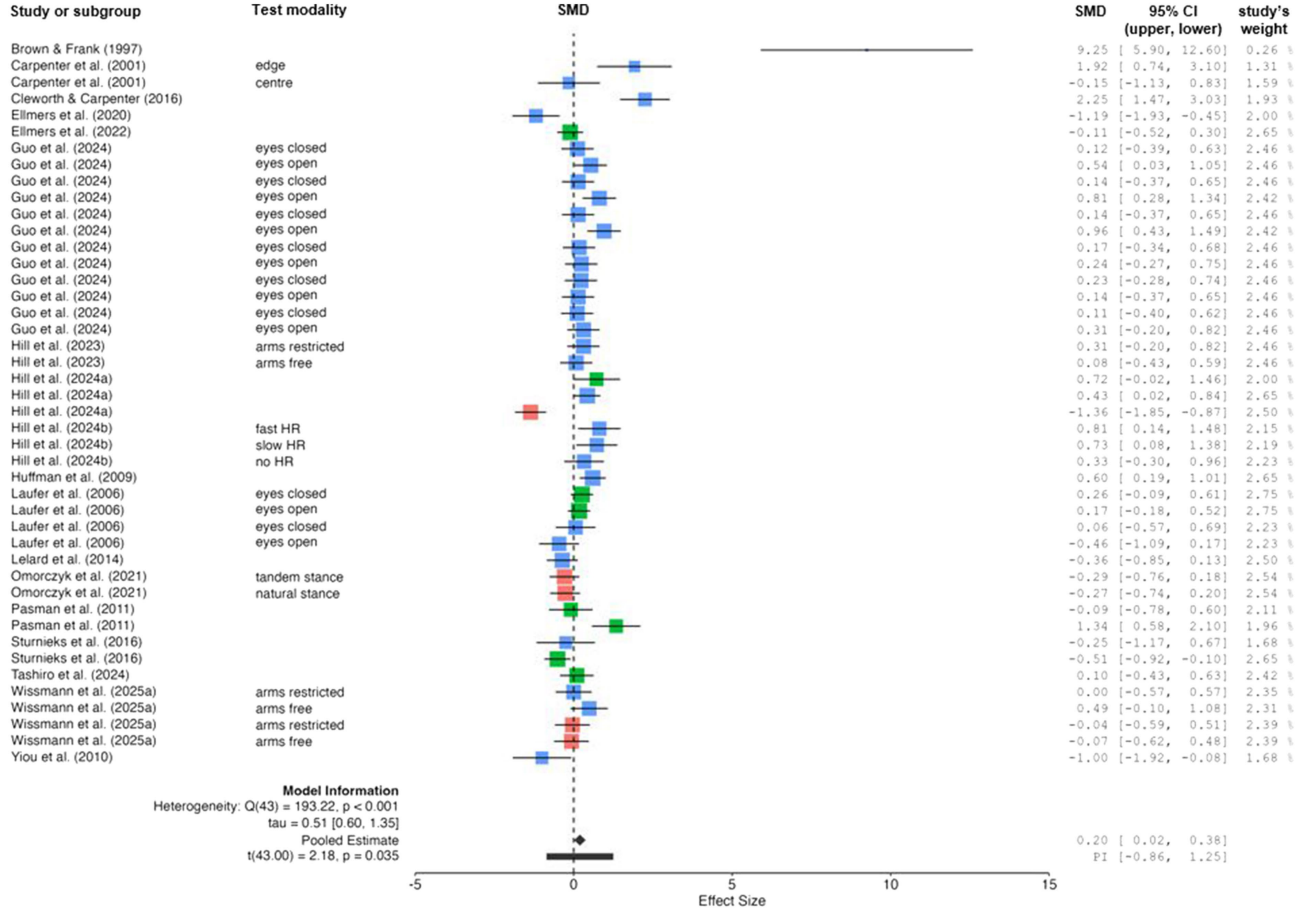
Changes in static balance performance (i.e., sway amplitude measures) by threat condition [i.e., no threat (ground-level) *vs.* threat (above ground-level)]. Studies displayed in red, blue, and green are indicative for children, young adults, and older adults, respectively. CI confidence interval, HR heart rate, PI prediction intervals, SMD standardized mean difference.

The comparison of changes in static balance performance (i.e., sway amplitude measures) between ground (no threat) and height (threat) conditions is shown in Figure 3. The weighted mean *SMD* value amounted to 0.20 (18 studies, 44 comparisons) indicating a positive and small effect. Overall heterogeneity between studies ranged from substantial to considerable (*I*^2^ = 77%). Age-specific subgroup analyses revealed a reduction in heterogeneity for young (*I*^2^ = 70%) and older (*I*^2^ = 70%) adults but not for children (*I*^2^ = 84%). The subsequently performed LOO method led to a further reduction in heterogeneity for young (*I*^2^ = 60% when the study of Brown and Frank (Brown and Frank, 1997) was excluded) and older [*I*^2^ = 53% when the study of Pasman et al. (2011) was excluded] adults. However, the LOO method could not be used for children because only three studies (Omorczyk et al., 2021; Hill et al., 2024a; Wissmann et al., 2025a) were available in total.

**Figure 4:**
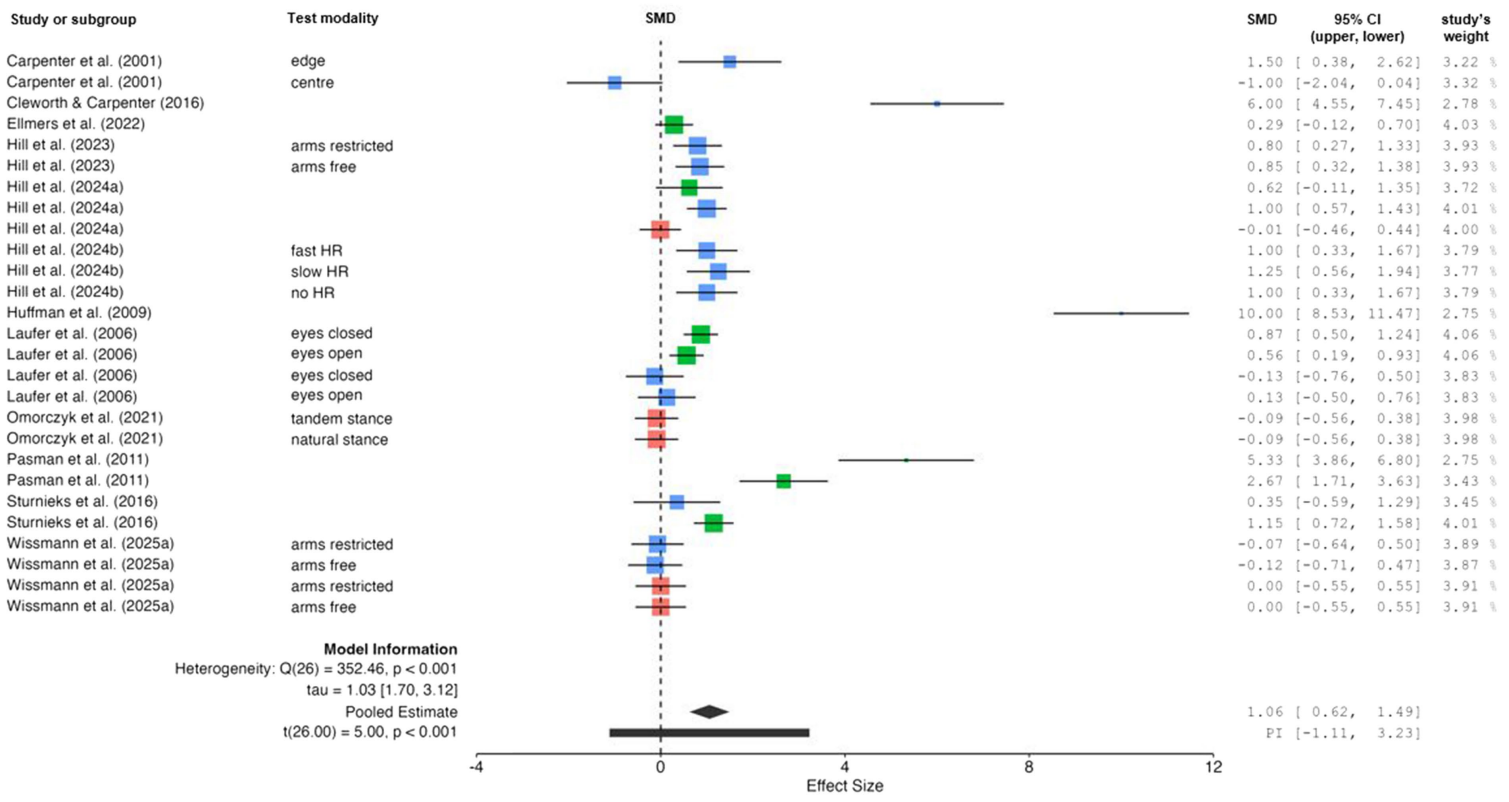
Changes in static balance performance (i.e., sway frequency measures) by threat condition [i.e., no threat (ground-level) *vs.* threat (above ground-level)]. Studies displayed in red, blue, and green are indicative for children, young adults, and older adults, respectively. CI confidence interval, HR heart rate, PI prediction intervals, SMD standardized mean difference.

Figure 4 illustrates the comparison of changes in static balance performance (i.e., sway frequency measures) between ground (no threat) and height (threat) conditions. The weighted mean *SMD* value amounted to 1.06 (12 studies, 27 comparisons) that is indicative for a positive and large effect. Overall heterogeneity between studies was considerable (*I*^2^ = 92%). Age-specific subgroup analyses showed a reduction in heterogeneity for children (*I*^2^ = 0%) but not for young (*I*^2^ = 94%) and older (*I*^2^ = 90%) adults. However, the LOO method revealed a reduction in heterogeneity for young [*I*^2^ = 66% when the study of Huffman et al. (2009) was excluded] and older [*I*^2^ = 57% when the study of Pasman et al. (2011) was excluded] adults. Both static balance measures showed a positive sign that indicate a potentially effective “stiffening” response (i.e., decreased sway amplitude and increased sway frequency).

### 3.4 Effect of height-induced postural threat on dynamic balance performance

**Figure 5:**
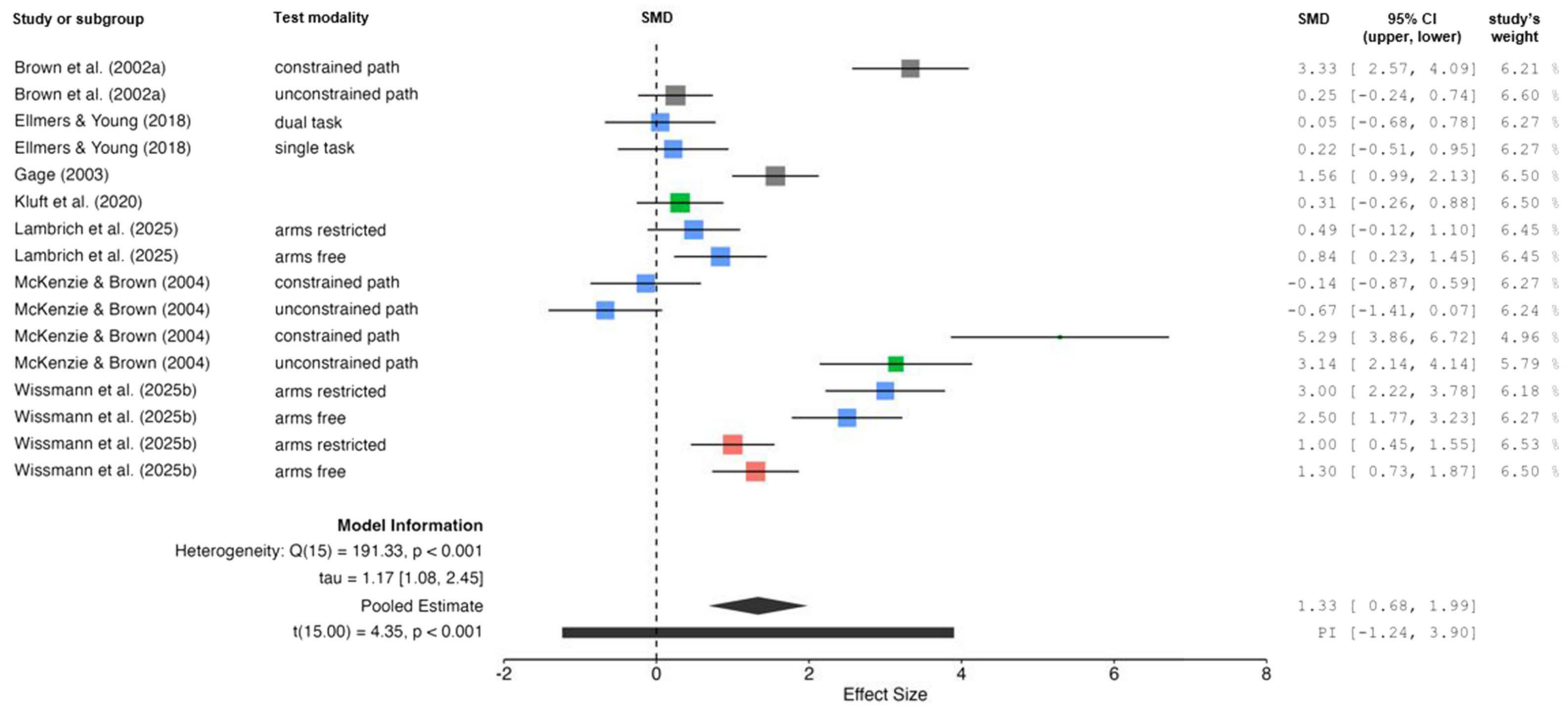
Changes in dynamic balance performance (i.e., gait velocity) by threat condition [i.e., no threat (ground-level) *vs.* threat (above ground-level)]. Studies displayed in red, blue, and green are indicative for children, young adults, and older adults, respectively. Gray indicates data that includes multiple age groups, as data for individual subgroups was not available. CI confidence interval, PI prediction intervals, SMD standardized mean difference.

The comparison of changes in dynamic balance performance (i.e., gait velocity) between ground (no threat) and height (threat) conditions is displayed in Figure 5. The weighted mean *SMD* value amounted to 1.33 (7 studies, 16 comparisons) indicating a positive and large effect. Overall heterogeneity between studies was considerable (*I*^2^ = 92%). Separate analyses for each age group revealed a reduction in heterogeneity for young (*I*^2^ = 48%) and older (*I*^2^ = 83%) adults. The LOO method could not be performed for older adults because only three studies (McKenzie and Brown, 2004; Brown et al., 2002a; Gage et al., 2003) with two comparisons were available.

### 3.5 Effect of height-induced postural threat on static and dynamic balance performance: role of task difficulty

The comparison of changes in static and dynamic balance performance between ground (no threat) and height (threat) conditions depending on the level of task difficulty (e.g., standing with eyes open *vs.* closed or single-task *vs.* dual-task walking) included 13 studies (Carpenter et al., 2001; Brown et al., 2002a; Gage et al., 2003; McKenzie and Brown, 2004; Laufer et al., 2006; Ellmers and Young, 2018; Omorczyk et al., 2021; Guo et al., 2023; Hill et al., 2023; Hill et al., 2024b; Lambrich et al., 2025; Wissmann et al., 2025b; a) revealed that when the level of task difficulty increased, the *SMD* value increased for dynamic (low: *SMD* = 1.05 *vs.* high: *SMD* = 1.78) and partially for static (sway frequency, low: *SMD* = 0.23 *vs.* high: *SMD* = 0.43; sway amplitude, low: *SMD* = 0.24 *vs.* high: *SMD* = 0.18) balance performance. According to this finding, an increase in task difficulty led to greater effects in terms of a reduction in gait speed and sway frequency measures but did not change sway amplitude outcomes.

### 3.6 Effect of height-induced postural threat on static and dynamic balance performance: age differences

Regarding static balance, age-specific sub-analyses included four studies (Laufer et al., 2006; Sturnieks et al., 2016; Hill et al., 2024a; Wissmann et al., 2025a) and yielded that children compared to young and older adults adopted a different postural adaptation strategy when standing at height. Specifically, adults showed positive *SMD* values (sway amplitude, young adults: *SMD* = 0.11, old adults: *SMD* = 0.11; sway frequency, young adults: *SMD* = 0.21, old adults: *SMD* = 0.82) but children displayed negative *SMD* values (sway amplitude: *SMD* = − 0.50; sway frequency: *SMD* = −0.01). The former are indicative of potentially protective “stiffening” response, and the latter implies an insufficiently adapted and ineffective postural adaptation strategy. In terms of dynamic balance, no *SMD* values could be calculated because only one study (Wissmann et al., 2025b) directly compared different age groups (children vs. young adults) when walking on ground and at height. In addition to child–adult comparisons, three studies directly compared young and older adults under dynamic conditions (Brown et al., 2002a; Gage et al., 2003; McKenzie and Brown, 2004). Older adults generally showed greater reductions in gait velocity and increased double-support duration at height compared to young adults.

## 4 Discussion

Building on a previous narrative review (Adkin and Carpenter, 2018), this systematic review with meta-analysis characterized, summarized, and quantified the effects of height-induced postural threat with varying levels of task difficulty on static and dynamic balance across different age groups. Meta-analysis of 25 studies including 877 participants (*n* = 459 females) revealed that both young and older adults adapted their balance (e.g., by reducing sway amplitude and increasing sway frequency) and gait (e.g., by slowing their walking speed) behavior at height in ways that likely serve to increase stability. In contrast, children did not show these adaptive responses, suggesting that their balance adjustments to height-induced postural threats were insufficiently adapted (i.e., a strategy that allowed the CoM to move closer toward the BoS). The results further indicate that task difficulty influenced balance responses, that is, height-induced postural threat was more pronounced during challenging conditions, particularly for dynamic balance tasks like walking.

### 4.1 Effects of height-induced postural threat on balance performance

In addition to previous original studies (Adkin et al., 2008; Carpenter et al., 2001; Hill et al., 2023), our meta-analysis provides robust empirical evidence that height-induced postural threat elicits distinct balance adaptations in adults, characterized by a potentially protective “stiffening” response. This manifests as a reciprocal reduction in sway amplitude (*SMD* = 0.20) and an increase in sway frequency (*SMD* = 1.06) during static balance tasks and likely reflects a CNS-mediated response to minimize movements of the CoP when the perceived consequences of falling are heightened (Adkin and Carpenter, 2018). The more pronounced changes in sway frequency compared to sway amplitude implies that height-induced threats primarily drive tighter, rapid postural adjustments to control CoM rather than simply restricting overall movement. This finding aligns with the theoretical framework proposing that increased co-contraction of antagonist muscle groups provide more joint stability, enabling finer neuromuscular control to keep the CoM securely within the BoS (Warnica et al., 2014). The relatively modest and inconsistent reductions in sway amplitude reported across the literature should be interpreted in light of notable differences in the reported experimental protocols (i.e., height of threat and whether a safety harness was used). In this regard, standing/walking height ranged between 5 and 320 cm. In addition, seven out of 25 studies used a safety harness whereas the remaining 16 studies did not (Table 1). Further methodological discrepancies were observed with respect to individual variability in height-induced postural threat perception (i.e., due to age, generalized concerns about falls or attention to movement processes) and/or the possibility that some individuals adopt alternative balance control strategies (i.e., such as exploratory movements).

A further notable finding – that is consistent with previous original studies (Brown et al., 2002b; Ellmers and Young, 2018; Gage et al., 2003) was the substantial reduction in gait velocity when walking at height (*SMD* = 1.33). It is generally accepted that a slower gait velocity reflects a deliberate, more cautious gait pattern modification when balance safety is threatened, which likely serves as a compensatory mechanism to increase stability (i.e., increasing amount of time spent in double support) and reduces the risk of falls when navigating at elevated surfaces (Brown et al., 2002b; Delbaere et al., 2009; Ellmers et al., 2020a).

### 4.2 Effects of height-induced postural threat and the role of task difficulty

Our subgroup analyses revealed that task difficulty significantly mediates the relationship between height-induced postural threat and balance performance. That is, the effects were more pronounced under challenging balance task conditions. This interaction was particularly evident for dynamic balance, where the large effect size for reduced gait velocity under low difficulty task conditions (*SMD* = 1.05) substantially increased by 70% under high difficulty task conditions (*SMD* = 1.78). In this review, dynamic tasks with a low degree of difficulty included single-task walking, walking with free arm movements, walking on wide beams, while high difficulty tasks included dual-task walking, walking with restricted arm movements and walking on narrow beams. This may be attributed to the increased cognitive and physical demands evoked by more challenging dynamic tasks, which could exacerbate fear and anxiety responses and subsequently result in exaggerated postural adjustments (i.e., reductions in gait velocity; Granacher et al., 2011; Barbado Murillo et al., 2012; Gebel et al., 2019). In terms of static balance, the low-difficulty tasks involved standing in the center of a platform, with eyes open, arms free, or in a bipedal stance, while the high-difficulty tasks involved standing at the edge of a platform, with eyes closed, arms restricted, or receiving false feedback. The impact of task difficulty was more nuanced in static than in dynamic balance. For example, while sway frequency showed a greater increase under high versus low difficulty task conditions (high: *SMD* = 0.43 *vs.* low: *SMD* = 0.23), sway amplitude remained relatively unchanged regardless of task difficulty (high: *SMD* = 0.18 *vs.* low: *SMD* = 0.24). One interpretation for this pattern of results is that the “stiffening” response may have an upper threshold beyond which additional task constraints do not further reduce movement amplitude, though the frequency of corrective actions continues to increase to maintain stability (Adkin et al., 2008, Carpenter et al., 2001).

### 4.3 Effects of height-induced postural threat and the role of age

The most striking observation is the age-dependent variation in response to height-induced postural threat. While adults consistently demonstrated a “stiffening” response—characterized by decreased sway amplitude (young adults: *SMD* = 0.11, old adults: *SMD* = 0.11) and increased sway frequency (young adults: *SMD* = 0.21, old adults: *SMD* = 0.82) of the CoP—children exhibited markedly different patterns, with increased sway amplitude (*SMD* = −0.50) and negligible changes in sway frequency (*SMD* = −0.01). Although this pattern of postural control, involving larger but slower movements of the CoP, could be interpreted as a more ‘relaxed’ strategy, we argue that it is insufficiently adapted (Hill et al., 2024a), as it appears reckless in the context of standing at height. Specifically, the large excursions of the CoM toward the limits of stability heighten the risk of losing balance. We interpret the absence of a protective “stiffening” response in children as evidence of fundamental differences in how their developing the nervous system process height-induced threat-related information and implement motor control strategies. The divergent behavior detected in children compared to adults is most likely due to maturational deficits in the ability to control posture in children (Forssberg and Nashner, 1982; Shumway-Cook and Woollacott, 1985; Foudriat et al., 1993; Hirabayashi and Iwasaki, 1995; Orendorz-Fraczkowska and Kubacka, 2019; Sinno et al., 2021) and emotional processing capacity (De Sonneville et al., 2002; Perlman and Pelphrey, 2011). For example, Shumway-Cook and Woollacott (Shumway-Cook and Woollacott, 1985) applied moving platform posturography and tested children of different age groups and adults under altered sensory conditions. While standing with eyes closed (i.e., proprioception dominant), children aged 4–6 years swayed more than 7- to 10-year-olds and adults. When the support surface was servoed (i.e., visual input dominant), postural sway further increased in 4- to 6-year-old children compared to those aged 7–10 years and adults. When combining this condition with closed eyes (vestibular dominant), postural sway even further increased in children aged 4–6 years and was higher than in 7- to 10-year-olds and adults. Within this context, Hirabayashi and Iwasaki (Hirabayashi and Iwasaki, 1995) used the Sensory Organization Test and examined children of different ages (3–15 years) and adults. They showed that proprioceptive function developed early, visual function followed and reached the adult level at the age of 15 years. The vestibular function developed later and showed a considerably lower level compared to adults even at the age of 15 years. These collective studies reveal that there are marked developmental changes with respect to postural control in grown-ups compared to adults.

It is also possible that the discrepancy between children and adults arises from the fact that our meta-analysis relies on only three studies on this topic (Wissmann et al., 2025a, 2025b; Hill et al., 2024a). Nonetheless, the inclusion of data on children highlights a developmental trajectory in height-induced threat-adaptive balance control that has not been previously emphasized in the literature. However, this remains a preliminary finding and future research aimed at replicating and expanding on this result would therefore be valuable.

Age differences between young and older adults were evident in both static and dynamic balance tasks. During static stance, older adults exhibited smaller sway amplitudes, but higher sway frequencies than young adults (Laufer et al., 2006; Sturnieks et al., 2016; Hill et al., 2024a), indicating a “stiffening” response that favors stability over exploratory postural adjustments. In dynamic gait tasks, older participants demonstrated slower walking velocities, longer double-support durations, and shorter step lengths when exposed to height (Brown et al., 2002a; Gage et al., 2003; McKenzie and Brown, 2004). These findings suggest that aging amplifies the conservative adaptations typically observed under postural threat, likely reflecting reduced sensory integration, diminished neuromuscular adaptability, and a heightened perception of threat. Collectively, older adults prioritize stability and safety, whereas younger adults maintain more flexible balance strategies under height-induced postural threat.

Limitations of this review include the absence of protocol registration in a public database such as PROSPERO. Future reviews should aim for prospective registration to enhance transparency and minimize risk of bias. In addition, the applied methodological approach varied between the included studies with respect to participants’ characteristics (i.e., chronological age, distribution of gender), height level (i.e., 5–320 cm), assessment (i.e., test characteristic, safety harness), and test modality (e.g., type of task difficulty) which is reflected in substantial to considerable heterogeneity between studies and were attempted to resolve by age-specific subgroup analyses and the application of the LOO method. Such variability limits generalizability and should be considered when interpreting the findings. Furthermore, age differences could not be calculated for measures of dynamic balance due to a lack of studies that directly compared different age groups and should therefore be investigated in future studies. Finally, it is important to acknowledge that other postural threat paradigms have been used to evoke negative emotions using support-surface translations, unstable platforms, or visual-flow perturbations, all of which can evoke fear and alter balance control (Carpenter et al., 2001; Cleworth and Carpenter, 2016). Therefore, the observations reported here should only be generalized to height-induced postural threat manipulations.

## 5 Conclusion

The analysis revealed that for static balance, height-induced postural threat led to small magnitude decreases in sway amplitude and large magnitude increases in sway frequency, indicative of a potentially effective “stiffening” response. However, age-specific analyses revealed that children showed an insufficiently adjusted postural adaptation strategy opposite to the “stiffening” response observed in adults when standing at height. Regarding dynamic balance, walking at height resulted in large magnitude reductions in gait velocity, with more pronounced effects observed under high compared to low task difficulty conditions. Like children, older adults exhibited greater conservative adaptations to height than young adults, indicating age-dependent modulation of balance control under threat. During static balance tasks, older adults showed reduced sway amplitude but increased sway frequency, reflecting a “stiffening” response that enhances stability at the expense of flexibility. In dynamic gait tasks, elderly adopted slower walking velocities, longer double-support durations, and shorter step lengths—signs of a cautious locomotor strategy aimed at minimizing fall risk. These age-related patterns suggest that older adults prioritize stability and safety across both standing and walking conditions, whereas young adults maintain more adaptive and exploratory control strategies under height-induced postural threat. Future research should focus on (1) directly comparing age groups during dynamic balance tasks, (2) further investigating the preliminary findings regarding children’s insufficiently adapted responses to height-induced postural threat, and (3) exploring how different types of postural threat (e.g., support-surface translations, unstable platforms, or visual-flow perturbations) influences balance outcomes.

## Data Availability

The original contributions presented in the study are included in the article/Supplementary material, further inquiries can be directed to the corresponding author/s.
